# “I’d like more options!”: Interviews to explore young people and family decision-making needs for pain management in juvenile idiopathic arthritis

**DOI:** 10.1186/s12969-023-00849-0

**Published:** 2023-07-26

**Authors:** Karine Toupin-April, Isabelle Gaboury, Laurie Proulx, Adam M. Huber, Ciarán M. Duffy, Esi M. Morgan, Linda C. Li, Elizabeth Stringer, Mark Connelly, Jennifer E. Weiss, Michele Gibbon, Hannah Sachs, Aditi Sivakumar, Alexandra Sirois, Emily Sirotich, Natasha Trehan, Naomi Abrahams, Janice S. Cohen, Sabrina Cavallo, Tania El Hindi, Marco Ragusa, France Légaré, William B. Brinkman, Paul R. Fortin, Simon Décary, Rebecca Lee, Sabrina Gmuca, Gail Paterson, Peter Tugwell, Jennifer N. Stinson

**Affiliations:** 1 School of Rehabilitation Sciences, Faculty of Health Sciences, University of Ottawa, Ottawa, ON Canada; 2 Department of Pediatrics, Faculty of Medicine, University of Ottawa, Ottawa, ON Canada; 3 Children’s Hospital of Eastern Ontario Research Institute, Ottawa, ON Canada; 4 Institut du Savoir Montfort, Ottawa, ON Canada; 5 Department of Family and Emergency Medicine, Faculty of Medicine and Health Sciences, Université de Sherbrooke, Longueuil, Québec Canada; 6 Canadian Arthritis Patient Alliance, Ottawa, ON Canada; 7 Division of Rheumatology, IWK Health Centre, Halifax, NS Canada; 8 Department of Pediatrics, Dalhousie University, Halifax, NS Canada; 9 Department of Pediatrics, Faculty of Medicine, Ottawa, ON Canada; 10 Division of Rheumatology, Seattle Children’s Hospital, Seattle, Washington USA; 11 Department of Pediatrics, University of Washington School of Medicine, Seattle, WA USA; 12 Department of Physical Therapy, Faculty of Medicine, University of British Columbia, Vancouver, BC Canada; 13 Arthritis Research Canada, Vancouver, BC Canada; 14 Department of Pediatrics, Children’s Mercy Kansas City, Kansas City, MO USA; 15 Division of Rheumatology, Hackensack University Medical Center, Hackensack Meridian School of Medicine, Hackensack, NJ USA; 16 Public Health Agency of Canada, Ottawa, ON Canada; 17 Queen’s University, ON Ottawa, Canada; 18 Canadian Arthritis Patient Alliance, ON Sudbury, Canada; 19 Canadian Arthritis Patient Alliance, New Haven, CT USA; 20 Yale University School of Medicine, New Haven, CT USA; 21 University of Ottawa, Ottawa, ON Canada; 22 Faculty of Social Sciences, University of Ottawa, Ottawa, ON Canada; 23 Behavioural Neurosciences and Consultation Liaison Team, Mental Health, Children’s Hospital of Eastern Ontario, Ottawa, ON Canada; 24 School of Psychology, Faculty of Social Sciences, University of Ottawa, ON Ottawa, Canada; 25 School of Rehabilitation, Faculty of Medicine, Université de Montréal, Montréal, QC Canada; 26 Centre for Interdisciplinary Research in Rehabilitation of Greater Montreal, Montréal, QC Canada; 27 Research Centre of the Sainte-Justine University Hospital, Montreal, QC Canada; 28 Statistics Canada, Government of Canada, Ottawa, ON Canada; 29 The Ottawa Hospital, University of Ottawa, Ottawa, ON Canada; 30 Department of Family and Emergency Medicine, Université Laval, Québec City, QC Canada; 31 VITAM, Centre de recherche en santé durable, Centre intégré universitaire de santé et services sociaux de la Capitale Nationale, Québec City, QC Canada; 32 Cincinnati Children’s Hospital Medical Center, OH Cincinnati, USA; 33 College of Medicine, Department of Pediatrics, University of Cincinnati, Cincinnati, OH United States; 34 Université Laval, QC, Canada; 35 CHU de Québec, Québec City, QC Canada; 36 School of Rehabilitation, Faculty of Medicine and Health Sciences, Université de Sherbrooke, Sherbrooke, QC Canada; 37 Research Center of the Centre Hospitalier de l’Université de Sherbrooke, Sherbrooke, QC Canada; 38 Centre for Epidemiology Versus Arthritis, Centre for Musculoskeletal Research, Division of Musculoskeletal and Dermatological Sciences, Faculty of Biology, Medicine and Health, University of Manchester, Manchester Academic Health Science Centre, Manchester, UK; 39 National Institute for Health Research Biomedical Research Centre, Manchester University Hospital NHS Trust, Manchester, UK; 40 Manchester Centre for Health Psychology, Division of Psychology and Mental Health, University of Manchester, Manchester, UK; 41 Division of Rheumatology, Children’s Hospital of Philadelphia, Philadelphia, PA USA; 42 Perelman School of Medicine, University of Pennsylvania, Philadelphia, PA USA; 43 Arthritis Rehabilitation and Education Program, Arthritis Society Canada, Ottawa, ON Canada; 44 School of Epidemiology and Community Medicine, Department of Medicine, University of Ottawa, Ottawa, ON Canada; 45 Clinical Epidemiology Program, Ottawa Hospital Research Institute, Ottawa, ON Canada; 46 WHO Collaborating Centre for Knowledge Translation and Health Technology Assessment in Health Equity, Bruyère Research Institute, Ottawa, ON Canada; 47 Child Health Evaluative Sciences, Research Institute, The Hospital for Sick Children, University of Toronto, Toronto, ON Canada; 48 Lawrence S. Bloomberg, Faculty of Nursing, University of Toronto, Toronto, ON Canada

**Keywords:** Juvenile idiopathic arthritis, Pain management, Decision-making needs, Shared decision making

## Abstract

**Background:**

Juvenile idiopathic arthritis (JIA) is a common pediatric rheumatic condition and is associated with symptoms such as joint pain that can negatively impact health-related quality of life. To effectively manage pain in JIA, young people, their families, and health care providers (HCPs) should be supported to discuss pain management options and make a shared decision. However, pain is often under-recognized, and pain management discussions are not optimal. No studies have explored decision-making needs for pain management in JIA using a shared decision making (SDM) model. We sought to explore families’ decision-making needs with respect to pain management among young people with JIA, parents/caregivers, and HCPs.

**Methods:**

We conducted semi-structured virtual or face-to-face individual interviews with young people with JIA 8–18 years of age, parents/caregivers and HCPs using a qualitative descriptive study design. We recruited participants online across Canada and the United States, from a hospital and from a quality improvement network. We used interview guides based on the Ottawa Decision Support Framework to assess decision-making needs. We audiotaped, transcribed verbatim and analyzed interviews using thematic analysis.

**Results:**

A total of 12 young people (*n* = 6 children and *n* = 6 adolescents), 13 parents/caregivers and 11 HCPs participated in interviews. Pediatric HCPs were comprised of rheumatologists (*n* = 4), physical therapists (*n* = 3), rheumatology nurses (*n* = 2) and occupational therapists (*n* = 2). The following themes were identified: (1) need to assess pain in an accurate manner; (2) need to address pain in pediatric rheumatology consultations; (3) need for information on pain management options, especially nonpharmacological approaches; (4) importance of effectiveness, safety and ease of use of treatments; (5) need to discuss young people/families’ values and preferences for pain management options; and the (6) need for decision support. Themes were similar for young people, parents/caregivers and HCPs, although their respective importance varied.

**Conclusions:**

Findings suggest a need for evidence-based information and communication about pain management options, which would be addressed by decision support interventions and HCP training in pain and SDM. Work is underway to develop such interventions and implement them into practice to improve pain management in JIA and in turn lead to better health outcomes.

## Significance and Innovations

This study highlights a need for information, communication and decision support about pain management options.

There is a need for assessing pain in an accurate manner and for sharing evidence-based information for pain management in JIA, especially for non-pharmacological treatment options.

There is a need for clarifying and discussing young people’s and families’ values and preferences about pain management in JIA and for a joint decision with HCPs.

Families’ values and preferences were similar to those of HCPs and included the importance of effectiveness, safety and ease of use of treatments.

Decision support interventions may enable HCPs to work with youth and families to address decision-making needs for pain management among young people with JIA.

### Background

Musculoskeletalpain is an important symptom of juvenile idiopathic arthritis (JIA) [1–3], with most young people experiencing some pain [1], and about 17% developing chronic pain [2]. Pain is associated with difficulties in physical, emotional, social and school functioning, thus affecting health-related quality of life and activity participation [4–10]. To effectively manage pain in JIA, a multi-disciplinary approach, including pharmacological, physical and psychological interventions, is required [11]. Unfortunately, pain in JIA is often under-recognized and communication about it is not optimal [12, 13].

An optimal way to make decisions, especially when there is no perfect treatment option and the choice depends on what families value most, is to engage in shared decision making (SDM). SDM is a process by which patients and health care providers (HCPs) make a joint decision by considering the best available evidence for treatment options as well as the patient’s and family’s values and preferences [14]. SDM is recommended as part of JIA treatment-to-target recommendations [15]. When supported by decision support interventions, SDM can lead to improved knowledge of treatment options, decisions which are consistent with patients’ values, and increased patient participation in decision-making [16].

Studies in JIA have shown that decision-making is not optimal [17, 18], but most studies focused on pharmacological disease management and not pain management, which often involves both pharmacological and non-pharmacological options [17]. Some studies revealed a lack of information-sharing with families on pain management options [17, 18], but other aspects of SDM have not been assessed thoroughly [17]. No study has assessed decision-making needs for pain management in the context of JIA using a SDM conceptual framework. We sought to explore families’ decision-making needs with respect to pain management among young people with JIA, parents/caregivers and HCPs.

## Methods

Reporting is based on the Standards for Reporting Qualitative Research (SRQR) reporting guidelines [19].

### Study design

We performed a decisional needs assessment based on the Ottawa Decision Support Framework (ODSF) [20] and on the workbook “Decisional Needs Assessment in Populations” [21]. Following a qualitative descriptive study design [22–24], we conducted semi-structured individual interviews with young people with JIA, their parents/caregivers and HCPs. Upon consent/assent, we conducted one interview per participant either face-to-face or online between October 2017 and August 2018. Interviewers audio-recorded interviews and took notes. The Children’s Hospital of Eastern Ontario (CHEO) Research Ethics Board approved this study (REB#16/100X) and participants signed consent/assent forms.

The research team comprised of women and men who are patient partners (LP, ASirois, ESirotich, NT, NA), clinicians from six professions (medicine, nursing, occupational therapy, physical therapy, psychology, social work), researchers, research coordinators and trainees, with varying level of familiarity with JIA pain management. Interviewers were experienced and trained in qualitative research. Interviewers explained the research goals to participants, their role and that there were no wrong answers. Interviewers had met some of the participants beforehand in other research projects.

### Sample

#### Young people and parents/caregivers

We recruited a purposive sample of young people aged 8 to 18 years old with JIA and parents/caregivers at the CHEO rheumatology clinic, as well as through the newsletter and social media of the Pediatric Rheumatology Care and Outcomes Improvement Network (PR-COIN), a learning health network of parents and clinicians in the United States and Canada (see selection criteria in Table 1). Purposive sampling was chosen so that participants’ characteristics varied in age, gender, disease severity and experience with pain management options. Eligible young people and parents/caregivers participated in face-to-face or online interviews with an interviewer (KTA, scientist, TEH or MG, research coordinators) and completed socio-demographic and medical information forms.

**Table 1:** Inclusion and exclusion criteria

	**Young people**	**Parents/caregivers**	**HCPs**
Inclusion criteria	Young people were eligible to participate in interviews if they: (a) were 8–18 years of age; (b) had been diagnosed with JIA by a rheumatologist; (c) had experienced JIA pain at any time in the past; and (d) were able to read/speak English	Parents/caregivers were eligible to participate if they: (a) lived with a child or adolescent diagnosed with JIA and (b) were able to read/speak English	HCPs were included if they had treated young people with JIA for at least one year
Exclusion criteria	Young people were excluded if they had (a) cognitive impairments or (b) major co-morbid illnesses which precluded them from participating in interviews and completing questionnaires	Parents/caregivers were excluded if they had (a) cognitive impairments or (b) major co-morbid illnesses which precluded them from participating in interviews and completing questionnaires	HCPs were excluded if they had not treated young people with JIA for at least one year

#### HCPs

We invited a purposive sample of pediatric rheumatology HCPs who were practicing at CHEO and/or were part of PR-COIN via e-mail and newsletter to ask them to participate in an interview in person or online with an interviewer (KTA). We included HCPs from various professions and different experiences with chronic pain management (Table 1).

#### Interview guides and questionnaires

Interview guides (Tables 2 and 3) were based on the ODSF[20] and included questions about the pain experience, and questions modified from the Personal Interview Questions for Client Key Informants [21]. We pilot-tested interview guides with two patients with JIA and a rheumatology colleague. Socio-demographic and medical information forms asked about children’s age and gender, family income, parents’ level of education and cultural background, as well as disease-related information. Another form asked about HCPs experience, location and type of practice.

**Table 2:** Interview guide for parents/caregivers (same interview guide with simpler language for young people)

Questions
1. What has your child’s pain level been in the last month (where 0 means no pain and 100 means extreme pain) when performing regular activities? 2. Can you please describe what kind of pain your child has? 3. Can you please tell me which treatments you have used to deal with your child’s pain? 4. Can you explain their advantages, disadvantages and risks? (for the ones that were the most used) 5. Thinking about pain treatments, what are other options that your child has? 6. Can you explain how you made the decision for (list each treatment)? 7. How did you feel when choosing the treatments? 8. a) What was important to you when choosing the pain treatments? b) Did you talk about what was important to you with your doctor/nurse? Did your doctor/nurse ask you about that? 9. What kind of information did you get when choosing your child’s pain treatments? Where did you get that information? 10. Are there some things that made it difficult for you to choose your child’s pain treatments? 11. The next time your child experiences pain, how would you like to be involved in choosing the pain treatment? 12. a) The next time your child experiences pain, what kind of information would you like to help you choose a treatment? b) How do you want to get that information? 13. Thank you for taking the time to speak with us. Do you have other comments?

**Table 3:** Interview guide for HCPs

Questions
1. Can you please describe your patients’ experiences with pain in juvenile arthritis? 2. Can you please describe your experience treating pain in juvenile arthritis? 3. Thinking about pain management in JIA, what are the options that patients have? 4. Can you please describe which pain management treatments you have recommended? 5. Can you please describe how pain management decisions are usually made in juvenile arthritis (*i.e.,* how pain treatments are chosen)? 6. How do patients and their parents/caregivers feel when making this decision? 7. What is important to you when choosing treatments to manage your patients’ pain? Which benefits, risks and inconveniences are the most important to you? 8. Do you discuss your patients’ (and their parent/caregiver’s) values and preferences when choosing pain treatments? 9. What kind of information do you provide when pain management decisions are being made? Where do you get that information? 10. Are there factors that make it difficult for your patients and their parents/caregivers to choose a pain management treatment? If so, can you please describe them? 11. Are there factors that help your patients and their parents/caregivers choose a pain management treatment? If so, can you please describe them? 12. In an ideal world, can you please describe how you would like pain management decisions to be made in juvenile arthritis (*i.e.,* how pain treatments should be chosen)? 13. What kind of information do you think would be helpful to your patients and their parent/caregiver when they are choosing pain management treatments? 14. What kind of help do you think your patients and their parents/caregivers want when they are choosing pain management treatments?
15. Thank you for taking the time to speak with us. Do you have other comments?

#### Data analyses

**Fig. 1:**
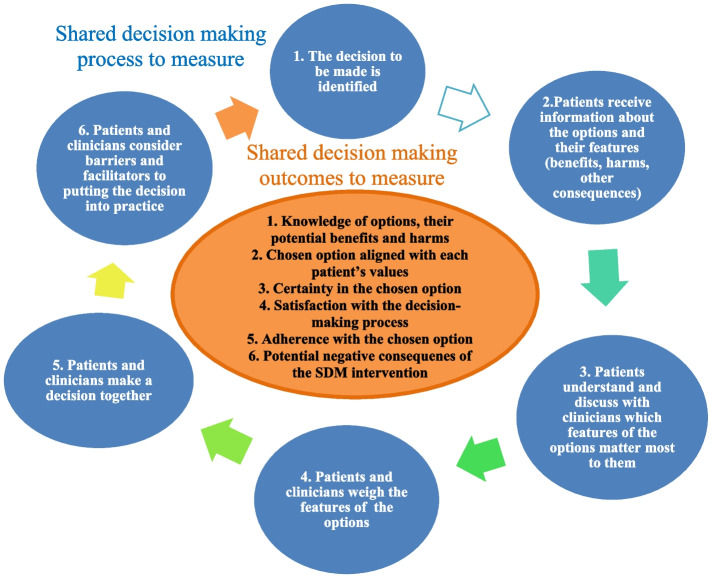
SDM process and outcomes to guide the analyses Fig. 1 shows the elements of the SDM process and outcomes used to guide the analyses

Audiotapes from the interviews and notes were transcribed verbatim. We analyzed the data using thematic analysis [25] with the help of the NVivo 11 software. Research trainees (DC, HS, ASivakumar, TEH, MR) coded and analyzed transcripts in pairs, and codes were discussed with KTA. We developed initial codes based on elements of the ODSF, and SDM process and outcomes, as per the Outcome Measures in Rheumatology (OMERACT) SDM Working Group work [26, 27] (Fig. 1). We added new emerging codes. We regrouped codes into overarching themes. We held team meetings and discussed findings to ensure these truly reflected participants’ experience rather than our own assumptions. We used an audit trail. We conducted interviews until data saturation was reached for each participant group, meaning that no new themes emerged as we conducted additional interviews, and we compared findings between groups. We analysed quantitative data using descriptive statistics in Statistical Package for the Social Sciences (SPSS;version 28).

## Results

### Participant characteristics

We approached 27 families. An additional 9 parents contacted the research team via social media. A total of 12 young people (*n* = 6 children and *n* = 6 adolescents) and 13 parents/caregivers (6 related to youths and 7 related to adolescents) participated in interviews. There were 12 young people/parent dyads and one additional parent/caregiver (Table 4).

**Table 4:** Young people and families’ disease-related and socio-demographic information

Characteristics	Young people included in dyads (*n* = 13)^a^
Age in years, median (range)	12 (8, 17)
Gender, n (%)	
Girl	11 (85)
Boy	2 (15)
JIA subtype, n (%)	(*n* = 10)^b^
Polyarticular	4 (40)
Oligoarticular	3 (30)
Psoriatic arthritis	1 (10)
Systemic	1 (10)
Enthesitis-related	1 (10)
Disease duration, years, median (range)	7 (1, 13)
Disease activity, n (%)	
Inactive disease	8 (62)
Active disease	5 (38)
Pain intensity in the past week, median (range)^c^ Young people report Parent report	(*n* = 11)^b^ 30 (3, 60) (n = 12)^b^ 25 (0, 45)
Country of residence, n (%)	
Canada	11 (85)
United States	2 (15)
Cultural background, n (%)^d^	(*n* = 12)^b^
Canadian	8 (67)
European	3 (25)
African	1 (8)
Asian	1 (8)
Parents’ level of education, n (%)	(*n* = 12)^b^
High school completed	1 (8)
College/Cegep	4 (33)
University	7 (58)
Family income (range), n (%)	(*n* = 11)^b^
Less than $14,999	1 (9)
$35,000-$44,999	1 (9)
$65,000-$74,999	1 (9)
$75,000-$84,999	2 (18)
$85,000-$94,999	3 (27)
More than $95,000	3 (27)

^a^Information on 13 young people from the dyads even if one of the young people did not participate in the interview

^b^ Some of the variables had missing data

^c^Visual analog scale 0–100, with 0: no pain, 100: worst pain

^d^Some participants identified with more than one cultural background

Some families declined participation (*n* = 3) or initially accepted but later declined (*n* = 20) because of their schedule. Out of 13 young people, 11 were girls and eight had inactive disease. Young people and parents reported a median pain value over the past week of 30 mm and 25 mm out of 100 mm, respectively. Participants reported currently using both medication and non-pharmacological options to manage pain (Table 5).

**Table 5:** Interventions currently used by young people

Interventions	Young people (*n* = 12)
Medication	
Non-steroidal anti-inflammatory drugs	10
Disease-modifying antirheumatic drugs	8
Biologics	6
Acetaminophen	4
Joint injection	3
Non pharmacological options	
Heat	10
Cold/ice	9
Rest	5
Stretching	5
Physical activity	5
Physiotherapy	5
Splints	3
Meditation	3
Massage therapy	3
Chiropractic	3
Supportive shoes	1
Brace	1
Distraction	1
Breathing	1
Hydrotherapy	1
Homeopathic cream	1

Eleven HCPs, comprising of pediatric rheumatologists (*n* = 4), physical therapists (*n* = 3), pediatric rheumatology nurses *(n* = 2) and occupational therapists (*n* = 2) participated (see Table 6).

**Table 6:** Health care providers’ clinical practice information

Characteristics	Health care providers (*n* = 11)
Country of work	
Canada	9
USA	2
Location of practice	
Hospital practice	9
Community practice	2
Type of practice	
Pediatric rheumatology practice only	3
Pediatric rheumatology and other unrelated populations	3
Mostly unrelated populations with a small pediatric rheumatology practice	3
Pediatric rheumatology and chronic pain practice	2
Pain management options recommended by HCPs	
*Pharmacological options*	
Getting the disease under control first	8
NSAIDs	7
Acetaminophen	5
Prednisone	2
Corticosteroid joint injections	2
Aspirin	1
*Non-pharmacological options*	
Heat	8
Stretching and strengthening exercises	7
Massage	5
Cold	5
Physical activity	5
Physiotherapy	4
Splints	4
Yoga	3
Meditation/mindfulness	3
Rest	3
Modify or avoid activities	3
Sleep hygiene	3
Technical devices	3
Braces	2
Apps (to help track symptoms and to relax)	2
Supportive shoes	2
Kinesiology tape/bandage	2
Occupational therapy	2
Joint protection	1
Relaxation	1
Diet	1
Tai chi	1
Foot orthotics	1
Acupuncture (if patient mentions it)	1
Transcutaneous Electrical Nerve Stimulation; TENS (if patient mentions it)	1
Coping skills	1
Digital pads	1
Referral to child life and social work	1
Referral to chronic pain clinic	1
Referral to mental health professional (e.g., psychologist)	1

### Themes

Findings revealed six themes which will be discussed. Quotes are also shown (see Table 7).

**Table 7:** Quotes illustrating decision-making needs

Themes	Quotes		
	Young people	Parents/caregivers	Health care providers
(1) Need to assess pain in an accurate manner	________	Difficulty for parents to assess their child’s pain: “So um, he doesn’t have a huge amount of pain. Um, sometimes he’s my funny child where sometimes you don’t know if it’s an excuse or whether it’s an actual thing. But then, you know, it will come out later that, no, no, the pain started before or something so it really was, you know, a pre-existing pain, rather than, oh you know, I was jogging and I turned my ankle or something right? Okay, but that was on Sunday, and you’re saying your ankle was hurting on Saturday right?” (P1, parent of a 12 year old) Difficulty for parent to accurately judge their child’s pain: (when parent is asked to describe the pain) “Um… It’s hard for me to do because I’m not her.” (P11, parent of a 15 year old) Easier for parents to assess their child’s pain as they get older: “As she’s gotten older though she’s better at telling us when she’s having good days and bad days and then, and then we can sort of modify her day rather than it impacting the whole day if that make sense.” (P3, parent of an 8 year old)	Difficulty assessing pain due to discrepancies between youths’ and parents’ reports: “…sometimes they will say “well my son has a lot of pain and he cannot function” and then you ask about “so what do you do for fun?”, “oh “I play hockey, basketball, this and that” and “how many times do you miss practice?”, “oh I never miss practice”. So, then I’m not sure… is the pain more a concern of the parent, thinking that there is pain?” (HCP1, rheumatologist) Difficulty assessing pain due to youths’ difficulty in remembering pain over time: “Sometimes if a child denies any pain, very often they come to a subsequent visit when the arthritis is better controlled and they’ll say something like, ‘Now I feel good’ or ‘Now I feel better’. They never mentioned the pain before because they couldn’t identify it but they do identify feeling much better.” (HCP 8, occupational therapist) Difficulty for youths to say they have pain and describe the type of pain, especially among younger youths: “I find that a lot of them don’t really complain about pain all that much… Or they… I mean they do when they’re asked, they admit they have pain. But they don’t volunteer a lot of that information. Especially because of their age… It’s very hard to explain pain even for an adult. So when kids talk about something hurting, they’re not very clear as to how to describe the type of pain. All they can say is it hurts.” (HCP 4, physical therapist)
(2) Need to address pain in pediatric rheumatology consultations;	________	Discussions with pediatric rheumatologists and nurses revolved mostly around disease activity and arthritis medication: “We’ve never been explained really any of the side effects [of pain medications which were added to biologics], we’ve been given [a page from the internet] and were told to “Google it”. When she’s been put on biologics they’ve taken a little bit more time and explained the side effects…” (P9, parent of a 10 year old) More in-depth discussion about pain management options when youths were referred to a chronic pain team: “If it wasn’t for our rheumatologist referring us [to the chronic pain clinic] I don’t think I would have gotten any information about how to manage her pain. Because they don’t give it to you in the rheumatology clinic you have to be referred to a chronic pain clinic before you get any kind of services or help about managing chronic pain.” (P9, parent of a 10 year old)	Rheumatologists and nurses mentioned that their first goal was to control disease activity: “…it's just our whole model of practice is very much driven by trying to control the disease." (HCP 2, rheumatologist) Interventions focused on medication to control disease activity, which would address the pain: “So usually we try to address the cause of the pain. So if they have pain because the knee is swollen, we use treatments to shrink the swelling and therefore alleviate the pain.” (HCP1, rheumatologist) Allied HCPs tended to focus on improving function: “…how to get them back functional to whatever, whatever they were doing before.” (HCP7, physical therapist)
(3) Need for information on pain management options, especially nonpharmacological approaches;	Participants know about only a few pain management options and need more information for non-pharmacological approaches: “Well sometimes I…I um I only have like three options that I go to: the stretching, the ice, the [naproxen], and I always am wondering, are there other treatments that I could take that aren’t going to affect my liver as much as [naproxen] will? Is there something that I can do that won’t have to do with medicine that I could just do that will provide me relief? […] Um, [I would like to learn] probably about dieting cause I know that that’s very important… And also psyche…like [psychology], because my doctor said that it’s all like how you think of it, how you’re feeling that day, what you’re putting into your mind, like, oh my pain hurts so bad…” (C4, 13 year old youth) Parents and youths were often unsure about the risks, side effects and benefits of options: (About the side effects of joint injections, a pharmacological treatment for active disease) “I don’t know about the risks but I know it’s supposed to work like better cause it’s directly in my knees.” (C2, 17 year old youth)	Discussion about medications but need more information on CHAs: “I would say that we’ve only really ever talked about medication with our medical team… we’ve never really had any um, um, like meaningful conversations about, about the alternative care.” (P3, parent of an 8 year old) HCPs provide just a few options: “They pretty much give you one or two choices and you’ve got to do the research on your own.” (P6, parent of a 11 year old) Parents and youths were often unsure about the risks, side effects and benefits of options: “I don’t think there is any risk going to a chiropractor. I really don’t. Um… Disadvantages I guess just time and money. That’s about it.” (P11, parent of a 15 year old) Need for evidence-based information and whether it worked for others for a range of options: “I know it’s like the “in” thing now. People are looking for other remedies, not just medication, but you know, like aroma therapy and all that. Like it’s not really research based, is it? I want those as options but at the same time, I also want to know… Are they research based? Are they proven? Is this going to help her or is it just a waste of money and a waste of time? It would be helpful to have all that information. Like you know, what are people saying works for them? Like if somebody’s used, I don’t know, aroma therapy on their child and it worked, I would like to know that cause I would want that as I would want to try it to see if it would work on my daughter, you know?” (P4, parent of a 13 year old)	HCPs mentioned that their lack of knowledge makes it difficult to discuss options with families: “I think we probably, as practitioners, don’t know necessarily about all of the options and so that becomes a challenge in terms of discussing all of these options with [families].” (HCP 2, rheumatologist) “We don’t really have any handouts to begin with. Um and the discussion is usually fairly brief. I would just say what the options are and I would say quite frankly that it depends on the patient what they end up using so they should try few of these and decide which one is most effective.” (HCP1, rheumatologist) Need for information on benefits and risks of non-pharmacological options: “I think a child who is in distress should have…families should have as much information as is available, right? Uh and to be done…to also have the information uh which includes the risks and the benefits I think is essential. We can't talk about an intervention that could harm a child without notifying the parents, as is done for drugs, among other things.” (HCP8, Occupational therapist)
(4) Importance of effectiveness, safety and ease of use of treatments;	Adolescents felt stigma when wearing splints: “Um, well splints for me, cause I’ve had to wear them in the past, um, I find that because I’m in high school, whenever I wear them, I get strange looks and I’m not really…I don’t really like that.” (C5, 16 year old)	Effectiveness is an important consideration when choosing pain management options: “We got to a point where I’ll… I’ll have her go on anything just to get rid of the pain” (P2, parent of a 17 year old) Worry about long-term adverse effects of medication and preference for youths to learn to manage their condition without pain medication: “Umm, I am concerned about like long term use [of Ibuprofen] and like stomach ulcers and things like that. Umm… I don’t want her to be dependent like feel like she has to reach for the medicine cupboard every time that she feels pain… Part of the arthritis is that pain comes along with that so like you can’t always…There’s not going to be a quick fix cure for it right? She has to learn to manage it.” (P3, parent of an 8 year old)	Preference to avoid pain medication for safety reasons: “I really don’t want patients on NSAIDs every day. I don’t think that it’s particularly safe.” (HCP9, rheumatologist) Treatment regimen should not overwhelm families: “I try not to put too much, not to clutter the family routine too much. So I don't put everyone on the same diet. It's the same for orthotics. The same for exercises. We'll only give what's necessary. I try to go to the minimum of what will bring a benefit that will make a difference in the life of the child. (HCP8, Occupational therapist)
(5) Need to discuss young people/families’ values and preferences for pain management options	HCPs usually told families to use pain medications without actively assessing their values and preferences: (When asked if they talked about their preferences with their HCPs) “They’re just kind of like, you know, this could help you… I was always told that this is going to help you, you’ll be able to do your normal functions…” (C5, 16 year old youth) Families accepted the treatment regimen but then pushed back: “… They have approached me with it, but we’d like usually me and my parents discuss it, we come back and, they understand that even though this is the better option, it doesn’t sound that it would really work…” (C5, 16 year old youth)	HCPs usually told families to use pain medications: “For [pain medication], it was there was no ifs and buts… No, it was, no she’s going to be on it for her pain and that’s it.” (P9, parent of a 10 year old) HCPs do not always respond to families’ preferences for non-pharmacological options: “…they aren’t very open to other ideas of pain management… Then really… It’s pretty much like twisting their arm. You know, this is my child. I don’t want her on that. She is not responding to this. You know I’ve looked online, I’ve talked to other parents, there are some other options out there can we try them? And after a while they see that the kid comes in month after month in pain and they’re like, “Okay, rather than increasing her medication, let’s try this”. And finally, you will win them over and you’ll get them to listen to you.” (P6, parent of a 11 year old)	HCPs do not ask directly about patient values and preferences: “I might do this indirectly through the conversation. I don’t think I ask this… like what’s your values about this. I don’t ask this directly. I think it’s something you get a sense of through the therapeutic relationship.” (HCP 3, nurse) HCPs do not spend enough time on discussing patient preferences in the consultation for pain management: “Uh I probably don’t spend enough time even though I just said I try to tailor my recommendations based on what they’re capable or willing to do. I mean I think I will generally lay some ideas out there and then step back and say are these reasonable. Um, unfortunately, I think most of the time patients will say yes and it isn’t until the follow-up visit that they’ll say no I didn’t do it at all that you missed a window of opportunity to come up with something better.” (HCP 10, rheumatologist)
(6) Need for decision support	Youths were actively engaged in choosing pain management options: “I usually tell them okay I need ice or I need a heating pad” (C10, 11 year old youth) Desire for tool to assess pain and provide evidence-based information on options and discussions with HCPs: “I like to discuss cause then it’s easier to ask questions… But it’s also really useful to have the information on paper or on a website… Cause when you have like, when you don’t remember you can still look at it and have the information. So I like both…Yes (it would be useful). And I would really like to have that for my phone… So if it hurts you can see what you can do at the moment… I like that.” (C2, 17 year old youth)	Youths were actively engaged in choosing pain management options: “Um, so then as far as massage goes, um, that is, if [patient’s name] says she’s in pain, I give her options: do you want to go to a massage? Do you want to go to the chiropractor? Do you want to do paraffin wax? And she decides which she wants to do. (P5, parent of a 16 year old youth) Desire for tool to provide evidence-based information on options: “I do wish there was some sort of a, um, a database or a…or some…a pamphlet or something that um, you know, listed ever…all the other, you know, options, you know, right down to the little things of the paraffin wax and the, and the massage therapy and the [Ibuprofen] and that kind thing. But really … we had to trial and error ourselves and um, so I do wish there was something that…that was at our fingertips that, you know, could suggest everything possible…” (P5, parent of a 16 year old youth)	Desire for tool to assess symptoms and provide evidence-based information on options: “I think if they had a tool to use to say if you have these symptoms you could do this, or if you have these particular symptoms you could do that… Some kind of tool that could give them various scenarios like you know this is what this is used for, that is what that is used for. These are the side effects it would help them a lot I think if they saw it written down… So certainly [we have to provide information for] physical [approaches], yes. Nutritional [approaches], yes… If they were having a lot of anxiety over the pain, or they were obviously sad, they might benefit from some psycho-social intervention… I think they need to know what’s out there.” (HCP 7, physical therapist)

### (1) Need to assess pain in an accurate manner

#### Young people and parents/caregivers

Some parents mentioned it was difficult for them to assess their child’s pain since they were relying on their child’s self-report which they did not always feel was accurate. Parents did not feel that their own perceptions of their child’s pain were accurate, and thus needed a better means to assess their child’s self-reported pain. One parent shared that a HCP did not think that their child’s pain was real. Parents felt that assessing pain was easier as youths became older.

### HCPs

Consistent with parents’ reports, HCPs described difficulty assessing youths’ pain. HCPs indicated this was due partly to discrepancies between youths’ and parents’ report of pain, and between reported pain and participation in their daily activities, as well as the youths’ difficulty in remembering pain over time, especially among younger children and those who do not experience a flare. A few HCPs mentioned not always assessing pain if families do not raise the issue.

A few HCPs reported that pain is not the predominant issue that families bring forward. HCPs thought that some young people may be reluctant to mention their pain to avoid escalating treatment. A few HCPs stated that even when young people report pain, they do not volunteer a lot of information about it, especially younger youths. Similar to parents, HCPs mentioned that youths, especially younger ones, have difficulty identifying and describing pain. A few HCPs voiced that pain is very subjective and cannot be predicted by physical findings. HCPs felt the need to assess the child’s self-report of pain and its functional impact in an accurate manner.

### (2) Need to address pain in pediatric rheumatology consultations

#### Young people and parents/caregivers

Young people and parents stated that discussions with pediatric rheumatologists and nurses revolved mostly around disease activity and arthritis medication (medication targeting disease remission). They had less thorough discussions specifically about pain and its treatments, including non-pharmacological options. Families mentioned that they discussed pain management options in more depth with HCPs, including a wider range of non-pharmacological options, when pain was the main disease feature and when they were referred to allied HCPs or a chronic pain team. Most felt that there is a need to address pain management in pediatric rheumatology.

### HCPs

Similar to young people’s and parents’ reports, rheumatologists mentioned that consultations do not usually focus on pain since it is usually not the dominant problem in JIA. They also mentioned having more thorough discussions about pain when youth have persistent pain with low disease activity.

Consistent with families’ responses, rheumatologists and nurses reported that their first goal was to control disease activity, which they felt should address the pain. Thus, their interventions focused on medication to control disease activity, followed by medication and non-pharmacological options to reduce residual pain. Allied HCPs and rheumatologists working in chronic pain clinics tended to focus more on symptom management (including pain) and improving function, which they achieved by discussing and recommending various options including a wider range of non-pharmacological options.

#### (3) Need for information on pain management options, especially non-pharmacological approaches

### Young people and parents/caregivers

Although most parents and young people mentioned receiving enough information on treatments targeting disease activity, most mentioned a need for more information on treatments specifically targeting pain, especially for non-pharmacological options and complementary health approaches (CHAs). They wished to receive information in a clear, concise and honest manner.

Participants reported that HCPs only presented a few pain management options when pain was an issue. They felt that some evidence-based information was presented on benefits and risks of medications but much less for non-pharmacological approaches. When asked, participants could identify a few potential pain management options, but were often unsure about benefits and risks, especially for non-pharmacological options.

Parents mentioned that information on pain management was provided at the time of diagnosis, but it was overwhelming. Also, they received information mostly in the consultation and were given little information between appointments. Parents mentioned they needed information throughout the disease course, as needed, with time to digest information between appointments.

Some parents and young people mentioned that HCPs provided information on pain management from pamphlets, books and health organisation websites. Many participants said they had to search for information on websites and social media or ask others, especially for non-pharmacological options. They found it useful to obtain information on various options and knowing what other parents tried but acknowledged that the information was not always reliable.

Overall, parents and young people wished to know more about a wide variety of pain management options, and scientific evidence of potential benefits and risks (short and long term), how well each worked for others, as well as logistics (e.g., time, cost), especially for non-pharmacological treatments. A few said that they wished to get probabilities of benefits and risks of treatments. They mentioned HCPs should be more educated on pain management, especially physicians and nurses concerning non-pharmacological options.

### HCPs

HCPs, especially physicians and nurses, voiced a lack of knowledge about available options for pain management and their evidence, mostly for non-pharmacologic options. Some also mentioned limited scientific evidence for these options, even though they did not search for it. They reported that this lack of knowledge makes it difficult to discuss the options with families, which is consistent with families’ perceptions.

Consistent with young people's and parents’ perspectives, most HCPs said they present benefits and risks with some evidence-based information to families but mostly for medications and less for non-pharmacological options. Some say they give a few options for non-pharmacological options that they have experience with, either professionally or personally. They mentioned that the information they provide on non-pharmacological options is not consistent and depends on each family’s needs and concerns.

HCPs mentioned sharing links to trusted websites to help guide families to manage pain. HCPs who treat more youth with chronic pain reported providing more resources such as handouts, websites and apps. HCPs acknowledged that families seek information online.

As with young people’s and parents’ wishes, HCPs suggested that families should receive information on a wider range of pain management options, especially non-pharmacologic options, and their benefits and risks. Information should be thorough but not overwhelm families.

### (4) Importance of effectiveness, safety, and ease of use of treatments

#### Young people's and parents/caregivers’ characteristics

Young people and parents reported that the most important consideration when choosing pain management options was treatment effectiveness. Most adolescents also wished to avoid injections because they are uncomfortable and splints because of stigma. It was also important for them and their caregivers to use treatments that were easy to use and did not disrupt their life.

Parents wished to avoid the potential short- and long-term side effects of pain medication. They preferred non-pharmacological options that would encourage their children to learn to manage their condition rather than relying on pain medication such as NSAIDs. Many parents felt that non-pharmacological options such as rest, heat, cold, stretching and deep breathing were helpful. However, some families mentioned lack of agreement between them and their child regarding the use of CHAs, with youths sometimes not interested in using these. Parents voiced practice variation among HCPs with some providers recommending non-pharmacologic interventions and others less so, with a lack of knowledge, belief or comfort with these interventions, especially CHAs.

### HCPs

Consistent with families’ perspective, HCPs acknowledged that families wished to avoid injections and preferred non-pharmacological treatments for fear of overmedicating; a sentiment echoed by most HCPs. Some HCPs preferred not to add pain medications because they have limited effectiveness and possible risks. Some mentioned that they did not always support the use of some non-pharmacological options because of their lack of knowledge. They mentioned that families should not be overwhelmed with a complex treatment regimen.

### (5) Need to discuss young people's/families’ values and preferences for pain management options

#### Young people and parents/caregivers

Young people and parents mentioned that HCPs listened to them and adjusted arthritis medications and additional pain medications based on their values. However, families reported that HCPs did not always actively ask about their values and preferences. HCPs usually recommended medications but changed them if families had issues. Some families said they had accepted the medical regimen but then discussed it at home and decided not to adopt the regimen.

For non-pharmacological approaches to help alleviate pain, there were few discussions with HCPs. When they happened, young people and parents mentioned that HCPs told them to use these options and then sometimes were asked if they wished to use these treatments and if it fitted in their life. A few parents and young people reported that HCPs do not always take into account their preferences for non-pharmacological options such as CHAs, but usually let them use these. Some mentioned that they had to bring new options and convince their HCPs. Parents and youths wished to discuss their values and preferences with HCPs.

### HCPs

Consistent with families’ perspective, some HCPs mentioned that they do not ask directly about patient values and preferences. They said that it usually emerges in the conversation over time as families ask questions about treatment options.

Some HCPs reported that they discuss patient preferences for pain management in the consultation but do not spend enough time on it. They tried to tailor information based on families’ concerns and willingness to use treatments. However, some mentioned that they often reached a decision with families but were then disappointed to see that they had not used the treatment.

### (6) Need for decision support

#### Young people and parents/caregivers

### Need for families and HCPs engagement in decision-making

Many families mentioned that HCPs often told them which treatment options to use and then families decided which treatments to use daily. Thus, many decisions to manage pain were made by families outside the clinical consultation. Parents and youths wished to be involved in decision-making by having a discussion with HCPs and felt current engagement in clinical settings is not optimal. Most parents mentioned that their youths, especially older ones, were actively engaged in choosing pain management options. They felt that this engagement is crucial since youths know their pain best and which options they could follow. Parents also wanted their youth to learn how to make decisions and have discussions with HCPs.

### Barriers to optimal decisions and their impact

Some parents and young people mentioned feeling pressured by their physician to use pain medication such as NSAIDs and felt they could not speak up. Parents sometimes felt pressured and criticized by friends for their treatment choices. Parents mentioned fear when their child was diagnosed and they had to choose medications.

Some young people felt stressed, confused and worried when choosing how to manage pain, especially at school, and unable to make a decision, especially regarding medications because of potential risks. Some youths felt upset about having to take pain medications.

Most participants mentioned being sure of their decision to manage pain but some acknowledged uncertainty because of their lack of knowledge, especially for non-pharmacological options. Most said they used the chosen options, but they sometimes stopped using treatments because of side effects they were not aware of. Families said that choosing non-pharmacological options was not based as much on their values as they wished. Some felt a lack of support to choose pain management options but mentioned that trusting HCPs facilitated decision-making.

### Need for decision support interventions and their potential impact

Parents and young people mentioned that having access to a tool to help assess pain and provide evidence-based information on options that match with their values would help them make decisions and engage in discussions with HCPs. Most families mentioned that the tool should be accessible on a computer/tablet/smart phone for easy access, and some wished to print the information. Most participants would like to use this tool between consultations and discuss information with HCPs using a printout or their phone.

Families felt that using an app or website would help ensure they have enough information and time to review and discuss with HCPs. Parents mentioned that giving information via an app developed by experts would help adolescents trust parents’ advice if recommendations are similar. Participants felt that using SDM would lead to more informed and personalized decisions, and youth empowerment.

### HCPs

#### Need for families and HCPs engagement in decision-making

HCPs reported that they typically recommended pain management options and let families determine how to use treatments on a daily basis. They mentioned engaging families and young people in discussions for pain management (with older youths being more engaged) but felt that families and young people should be more engaged in decision-making, especially for non-pharmacological options.

### Barriers to optimal decisions and their impact

HCPs mentioned that the lack of information and discussion about pain management led to uncertainty about which pain management options to use, especially non-pharmacological options. They also mentioned pressure on families from HCPs and others to use certain treatments. HCPs mentioned facilitators to decision-making such as the fact that families can voice their opinion. HCPs felt that families had more difficulty making decisions about arthritis medication because of potential risks. They felt that choosing how to deal with pain using non-pharmacological options was less difficult. A few HCPs mentioned that families were unsure which treatments to choose for pain after trying a few options.

#### Need for decision support interventions and their potential impact

HCPs reported that they would benefit from a tool to help describe young people’s pain and present evidence-based information on a range of treatments that match each young people’s values to engage in a collaborative approach to pain management. HCPs felt that an app or website that is easily accessible along with discussions with HCPs would be optimal to meet families’ needs. HCPs mentioned that some families may prefer an electronic version while others may prefer a paper version. HCPs also mentioned that using this tool could help them learn from families which options may be effective in real life.

## Discussion

This study identified families’ and HCPs’ mutually agreed upon decision-making needs related to JIA pain management. Findings reveal a need for assessing pain in an accurate manner and for sharing evidence-based information for pain management, especially on non-pharmacological treatment options. Participants also voiced a need for clarifying and discussing families’ values and preferences about pain management and for a joint decision with HCPs. Participants felt that a decision support intervention may enable HCPs to work with youth and families to better describe their pain and identify evidence-based treatment options to make informed and value-based decisions.

Findings emphasize a previously demonstrated need for assessing pain in an accurate manner [13, 18, 28]. Indeed, learning how to best recognize and treat pain is a research priority among youth with JIA [29]. However, studies have shown that it is difficult for parents and HCPs to accurately assess youths’ pain [13, 30] and HCPs are reluctant to assess pain, in part because of lack of training and confidence to do so [13]. Using validated tools, as suggested by our study and others [13, 18, 31], may address these barriers.

Findings reveal that consultations with HCPs do not often focus on pain management but mostly on disease activity, which is consistent with studies showing the low priority of addressing JIA pain [13]. This may explain the need for information on a wide range of pain management options for families [12, 32]. Allied HCPs and HCPs working in chronic pain clinics tend to discuss pain more often, its impact and a wide range of options, demonstrating the importance of specialized pain training [13]. Furthermore, information provided by HCPs varies and may not meet each family’s needs, thus showing the need to assess each family’s information needs [33]. Families must often resort to finding information online which, although not necessarily evidence-based, can be helpful [12, 34].

Several families’ values and preferences reflected those of HCPs and included the importance of effectiveness, safety and ease of use of treatments, which are similar to other studies [35, 36]. Families mentioned that HCPs wished to reduce pain medication and use non-pharmacological options, but that sometimes HCPs were not open to some options such as CHAs, showing that HCPs and families may not have the same preferences [33]. Parents also mentioned that their values and preferences are sometimes different from their children, which is consistent with studies on biologics in JIA and Crohn’s disease [37]. These differences among the youth-parent-HCP triad should be assessed and discussed to promote personalized decisions. Unfortunately, the current study shows that families and HCPs do not always discuss values and preferences which is similar to another study in JIA [38] and shows the need for a formal assessment of values and preferences.

Participants felt that families and young people should be engaged in decision-making about pain management and that current engagement is not optimal. There is a need to ensure optimal information-sharing and decision support to allow for decisions based on families’ and youths’ values and preferences. Participants felt a need for a tool to assess young people’s pain, provide evidence-based information on a range of pain management options and discuss families’ values and preferences in a joint decision with HCPs. Decision support interventions, such as patient decision aids (PDAs), decision coaching and HCP training in SDM, may be helpful in meeting these needs [16, 39–42].

### Study limitations

Most of the young people in our sample were girls, lived in Canada and had oligoarthritis or polyarthritis, which may preclude us from fully understanding the experience of boys, young people from the United States and those who have other JIA subtypes where pain is very common such as enthesitis-related arthritis. Also, we were not able to recruit all relevant HCPs, such as psychologists. However, we included psychologists in our research team and will engage them in developing decision support interventions.

## Conclusion

Patients with JIA, their caregivers and HCPs all identify a need to assess pain in an accurate manner and for sharing evidence-based information for pain management, especially on non-pharmacological treatment options. There is also a need for clarifying and discussing families’ values and preferences about pain management and for a joint decision with HCPs. A decision support intervention that addresses these needs, as well as HCP training about pain management and SDM, may enable HCPs to work with youth and families to better describe their pain and identify evidence-based treatment options to make informed and values-based decisions about pain management options. Work is underway to develop such interventions and implement them into practice to improve pain management in JIA and in turn lead to better health outcomes.

## Data Availability

The datasets analysed during the current study are not publicly available due to statements made in our Research Ethics Board application to protect anonymity of participants.

## Abbreviations

CHAs: Complementary health approaches
CHEO: The Children’s Hospital of Eastern Ontario
DMARDs: Disease-modifying antirheumatic drugs
JIA: Juvenile idiopathic arthritis
HCPs: Health care providers
NSAIDs: Non-steroidal anti-inflammatory drugs
ODSF: Ottawa Decision Support Framework
OMERACT: Outcome Measures in Rheumatology
PDAs: Patient decision aids
PR-COIN: Pediatric Rheumatology Care and Outcomes Improvement Network
SDM: Shared decision making
SPSS: Statistical Package for the Social Sciences
SRQR: Standards for Reporting Qualitative Research
TENS: Transcutaneous Electrical Nerve Stimulation

## References

[1] Bromberg MH, Connelly M, Anthony KK, Gil KM, Schanberg LE (2014). Self-reported pain and disease symptoms persist in juvenile idiopathic arthritis despite treatment advances: an electronic diary study. Arthritis Rheumatol.

[2] Rashid A, Cordingley L, Carrasco R, Foster HE, Baildam EM, Chieng A (2018). Patterns of pain over time among children with juvenile idiopathic arthritis. Arch Dis Child.

[3] Shiff NJ, Tupper S, Oen K, Guzman J, Lim H, Lee CH (2018). Trajectories of pain severity in juvenile idiopathic arthritis: results from the research in arthritis in canadian children emphasizing outcomes cohort. Pain.

[4] Schanberg LE, Anthony KK, Gil KM, Maurin EC (2003). Daily pain and symptoms in children with polyarticular arthritis. Arthritis Care Res.

[5] Haverman L, Grootenhuis MA, van den Berg JM, van Veenendaal M, Dolman KM, Swart JF (2012). Predictors of health-related quality of life in children and adolescents with juvenile idiopathic arthritis: results from a web-based survey. Arthritis Care Res.

[6] Cavallo S, Majnemer A, Mazer B, Chilingaryan G, Feldman DE (2015). Participation in leisure activities among canadian children with arthritis: results from a national representative sample. J Rheumatol.

[7] Yuwen W, Lewis FM, Walker AJ, Ward TM (2017). Struggling in the dark to help my child: parents’ experience in caring for a young child with juvenile idiopathic arthritis. J Pediatr Nurs.

[8] Tong A, Jones J, Craig JC, Singh-Grewal D (2012). Children’s experiences of living with juvenile idiopathic arthritis: a thematic synthesis of qualitative studies. Arthritis Care Res.

[9] Secor-Turner M, Scal P, Garwick A, Horvath K, Wells CK (2011). Living with juvenile arthritis: adolescents’ challenges and experiences. J Pediatr Health Care.

[10] Östlie IL, Johansson I, Möller A (2009). Struggle and adjustment to an insecure everyday life and an unpredictable life course: living with juvenile idiopathic arthritis from childhood to adult life–an interview study. Disabil Rehabil.

[11] Weiss JE, Luca NJC, Boneparth A, Stinson J (2014). Assessment and management of pain in juvenile idiopathic arthritis. Pediatr Drugs.

[12] Stinson JN, Toomey PC, Stevens BJ, Kagan S, Duffy CM, Huber A (2008). Asking the experts: exploring the self-management needs of adolescents with arthritis. Arthritis Care Res.

[13] Lee RR, Rashid A, Thomson W, Cordingley L (2020). “Reluctant to assess pain”: a qualitative study of health care professionals’ beliefs about the role of pain in juvenile idiopathic arthritis. Arthritis Care Res.

[14] Makoul G, Clayman ML (2006). An integrative model of shared decision making in medical encounters. Patient Educ Couns.

[15] Ravelli A, Consolaro A, Horneff G, Laxer RM, Lovell DJ, Wulffraat NM (2018). Treating juvenile idiopathic arthritis to target: recommendations of an international task force. Ann Rheum Dis.

[16] Stacey D, Légaré F, Lewis K, Barry MJ, Bennett CL, Eden KB (2017). Decision aids for people facing health treatment or screening decisions. Cochrane Database Syst Rev.

[17] Toupin April K, Laporte-Lafreniere M, Galibois G, Cadieux-Boileau G, Gaboury I, Grandpierre V, et al. A decision making needs assessment of youth with juvenile idiopathic arthritis and their caregivers: preliminary results from a narrative review. J Rheumatol. 2016;43:1201 (abstract).

[18] Stinson JN, Feldman BM, Duffy CM, Huber A, Tucker LB, McGrath PJ (2012). Jointly managing arthritis: information needs of children with juvenile idiopathic arthritis (JIA) and their parents. J Child Health Care.

[19] O’Brien BC, Harris IB, Beckman TJ, Reed DA, Cook DA (2014). Standards for reporting qualitative research: a synthesis of recommendations. Acad Med.

[20] Stacey D, Légaré F, Boland L, Lewis KB, Loiselle MC, Hoefel L (2020). 20th anniversary Ottawa decision support framework: part 3 overview of systematic reviews and updated framework. Med Decis Making.

[21] Jacobsen MJ, O’Connor AM, Stacey D, University of Ottawa. Decisional needs assessment in populations: a workbook for assessing patients’ and practitioners’ decision making needs. 1999. https://decisionaid.ohri.ca/docs/implement/population_needs.pdf. Accessed 3 Mar 2023.

[22] Sandelowski M (2000). Whatever happened to qualitative description?. Res Nurs Health.

[23] Sandelowski M (2010). What’s in a name?. Qualitative description revisited Res Nurs Heal.

[24] Kim H, Sefcik JS, Bradway C (2017). Characteristics of qualitative descriptive studies: a systematic review. Res Nurs Heal.

[25] Miles MB, Huberman AM, Saldana J. Qualitative data analysis: a methods sourcebook. 4th ed. Sage Publications; 2019.

[26] Toupin April K, Barton J, Fraenkel L, Li L, Grandpierre V, Guillemin F (2015). Development of a draft core set of domains for measuring shared decision making in osteoarthritis: an OMERACT working group on shared decision making. J Rheumatol.

[27] Toupin April K, Décary S, de Wit M, Meara A, Barton JL, Franenkel L (2021). Endorsement of the OMERACT core domain set for shared decision making interventions in rheumatology trials: results from a multi-stepped consensus-building approach. Semin Arthritis Rheum.

[28] Lovell DJ, Passo MH, Beukelman T, Bowyer SL, Gottlieb BS, Henrickson M (2011). Measuring process of arthritis care: a proposed set of quality measures for the process of care in juvenile idiopathic arthritis. Arthritis Care Res.

[29] Aussems K, Schoemaker CG, Verwoerd A, Ambrust W, Cowan K, Dedding C (2022). Research agenda setting with children with juvenile idiopathic arthritis: lessons learned. Child Care Health Dev.

[30] April KT, Feldman DE, Platt RW, Duffy CM (2006). Comparison between children with juvenile idiopathic arthritis (JIA) and their parents concerning perceived quality of life. Qual Life Res.

[31] Anthony KK, Schanberg LE (2005). Pediatric pain syndromes and management of pain in children and adolescents with rheumatic disease. Pediat Clin North Am.

[32] Tong A, Jones J, Speerin R, Filocamo K, Chaitow J, Singh-Grewal D (2013). Consumer perspectives on pediatric rheumatology care and service delivery: a qualitative study. J Clin Rheumatol.

[33] Lipstein EA, Brinkman WB, Sage J, Lannon CM, Morgan DE (2013). Understanding treatment decision making in juvenile idiopathic arthritis: a qualitative assessment. Pediatr Rheumatol.

[34] Lipstein EA, Lovell DJ, Denson LA, Moser DW, Saeed SA, Dodds CM (2013). Parents’ information needs in tumor necrosis factor-α inhibitor treatment decisions. J Pediatr Gastroenterol Nutr.

[35] Sanchez GAM. Case Western Reserve University. Parent and patient treatment preferences in juvenile idiopathic arthritis (JIA). Arthritis Rheum. 2012. https://etd.ohiolink.edu/apexprod/rws_etd/send_file/send?accession=case1323476963&disposition=inline. Accessed 4 Mar 2023.

[36] Anink J, Otten MH, Gorter SL, Prince FH, van Rossum MA, van den Berg JM (2013). Treatment choices of paediatric rheumatologists for juvenile idiopathic arthritis: etanercept or adalimumab?. Rheumatology.

[37] Lipstein EA, Dodds CM, Lovell DJ, Denson LA, Britto MT (2016). Making decisions about chronic disease treatment: a comparison of parents and their adolescent children. Health Expect.

[38] Lipstein EA, Dodds CM, Britto MT (2014). Real life clinic visits do not match the ideals of shared decision making. J Pediatr.

[39] Stacey D, Kryworuchko J, Bennett C, Murray MA, Mullan S, Légaré F (2012). Decision coaching to prepare patients for making health decisions: a systematic review of decision coaching in trials of patient decision aids. Med Decis Making.

[40] Légaré F, Stacey D, Turcotte S, Cossi MJ, Kryworuchko J, Graham ID (2014). Interventions for improving the adoption of shared decision making by healthcare professionals. Cochrane Database Syst Rev.

[41] Wyatt KD, List B, Brinkman WB, Lopez GP, Asi N, Erwin P (2015). Shared decision making in pediatrics: a systematic review and meta-analysis. Acad Pediatr.

[42] El Miedany Y, El Gaafary M, Lotfy H, El Aroussy N, Mekkawy D, Nasef SI (2019). Shared decision-making aid for juvenile idiopathic arthritis: moving from informative patient education to interactive critical thinking. Clin Rheumatol.

